# Cell signaling promoting protein carbonylation does not cause sulfhydryl oxidation: Implications to the mechanism of redox signaling

**DOI:** 10.12688/f1000research.11296.1

**Published:** 2017-04-10

**Authors:** Yuichiro J. Suzuki, Faisal Almansour, Camilla Cucinotta, Vladyslava Rybka, Lucia Marcocci

**Affiliations:** 1 Department of Pharmacology and Physiology, Georgetown University Medical Center, Washington, DC, 20057, USA; 2 Department of Biochemical Sciences “A. Rossi Fanelli”, Sapienza University of Rome, Rome, Italy

**Keywords:** cell signaling, protein oxidation, reactive oxygen species, redox signaling

## Abstract

Reactive oxygen species (ROS) have been recognized as second messengers, however, targeting mechanisms for ROS in cell signaling have not been defined. While ROS oxidizing protein cysteine thiols has been the most popular proposed mechanism, our laboratory proposed that ligand/receptor-mediated cell signaling involves protein carbonylation. Peroxiredoxin-6 (Prx6) is one protein that is carbonylated at 10 min after the platelet-derived growth factor (PDGF) stimulation of human pulmonary artery smooth muscle cells. In the present study, the SulfoBiotics Protein Redox State Monitoring Kit Plus (Dojindo Molecular Technologies) was used to test if cysteine residues of Prx6 are oxidized in response to the PDGF stimulation. Human Prx6 has a molecular weight of 25 kDa and contains two cysteine residues. The Dojindo system adds the 15 kDa Protein-SHifter if these cysteine residues are reduced in the cells. Results showed that, in untreated cells, the Prx6 molecule predominantly exhibited the 55 kDa band, indicating that both cysteine residues are reduced in the cells. Treatment of cells with 1 mM H _2_O _2_ caused the disappearance of the 55 kDa band and the appearance of a 40 kDa band, suggesting that the high concentration of H _2_O _2_ oxidized one of the two cysteine residues in the Prx6 molecule. By contrast, PDGF stimulation had no effects on the thiol status of the Prx6 molecule. We concluded that protein carbonylation is a more sensitive target of ROS during ligand/receptor-mediated cell signaling than sulfhydryl oxidation.

## Introduction

Reactive oxygen species (ROS) have been shown to play important roles in cell signaling ( Finkel, 2011; Suzuki *et al.*, 1997). In particular, the roles of ROS in cell growth signaling have been well documented ( Rao & Berk, 1992; Sundaresan *et al.*, 1995). For the mechanism of ROS signaling, the receptor activation producing ROS via NAD(P)H oxidase is a widely accepted concept ( Griendling *et al.*, 1994). However, molecular targeting mechanisms for ROS in cell signaling have been unclear. ROS targeting protein cysteine thiols has been the most popular proposed mechanism ( D’Autreaux & Toledano, 2007; Forman *et al.*, 2010; Moran *et al.*, 2001; Rhee *et al.*, 2000; Sen, 2000; Truong & Carroll, 2012; Veal *et al.*, 2007), yet the occurrence of thiol oxidation requires levels of ROS that are much higher than what is expected to occur during cell signaling ( Burgoyne *et al.*, 2007).

Our laboratory has proposed that ligand/receptor-mediated cell signaling involves protein carbonylation ( Wong *et al.*, 2008; Wong *et al.*, 2010), which occurs on four susceptible amino acid residues: proline, arginine, lysine, and threonine ( Amici *et al.*, 1989; Berlett & Stadtman, 1997). Notably, in cultured cells, hydrogen peroxide (H _2_O _2_) as low as 0.5 µM was found to promote protein carbonylation ( Wong *et al.*, 2008).

More recently, we identified proteins that are carbonylated in response to the platelet-derived growth factor (PDGF) stimulation. Among them, peroxiredoxin-6 (Prx6) was found to be carbonylated in response to a 10-min treatment of human pulmonary artery smooth muscle cells with PDGF ( Wong *et al.*, 2013). Peroxiredoxins have been shown to regulate cell signaling ( Woo *et al.*, 2010). The present study tested whether this signaling mechanism also promotes sulfhydryl oxidation within the Prx6 molecule.

## Methods

HPASMCs (ScienCell Research Laboratories, Carlsbad, CA, USA) were serum-starved overnight and treated with recombinant human PDGF-BB or H _2_O _2_ for 10, 15 or 30 min. Protein thiol states were monitored using SulfoBiotics Protein Redox State Monitoring Kit Plus (Dojindo Molecular Technologies, Rockville, MD, USA) in accordance with the manufacturer’s instructions. Briefly, cells were washed, proteins precipitated with trichloroacetic acid and “Protein-SHifters” were added to each sample. Samples were then loaded onto a sodium dodecyl sulfate polyacrylamide gel and electrophoresed. The gel was exposed to UV light to cut the “Protein-SHifters.” The resultant non-reducing SDS polyacrylamide gel was electroblotted to a nitrocellulose membrane (Bio-Rad Laboratories, Hercules, CA, USA). The membrane was blocked with 5% milk for 30 min at room temperature and incubated with the anti-Prx6 antibody produced in rabbit (Sigma-Aldrich Chemical Company, St. Louis, MO, USA; Catalogue no. P0058; 1:1,000 dilution) at 4°C overnight. The membrane was then washed three times and incubated with goat anti-rabbit IgG-horseradish peroxidase conjugate (Bio-Rad; Catalogue no. 1706515; 1:3,000 dilution) for 45 min at room temperature. After washing three times, signals were obtained using an Enhanced Chemiluminescence System (GE Healthcare Bio-Sciences, Pittsburgh, PA, USA).

## Results

**Figure 1.**
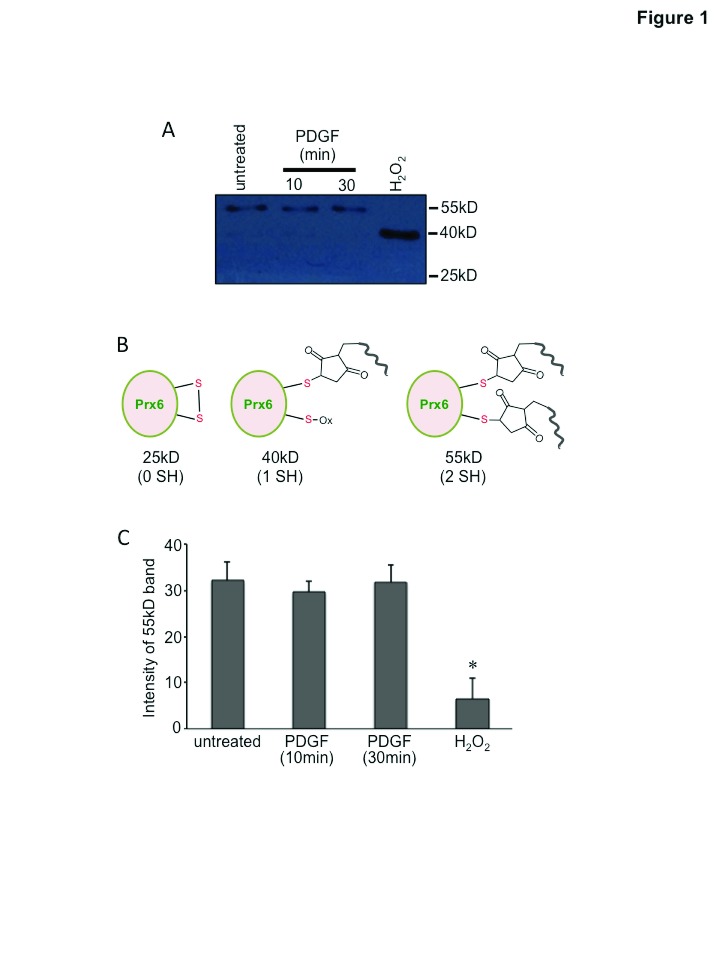
The thiol state of the Prx6 molecule is not altered by PDGF stimulation. Human pulmonary artery smooth muscle cells were treated with PDGF (10 ng/ml) for 10 or 30 min as described in Wong *et al.* (2013), or with H _2_O _2_ (1 mM) for 15 min. Cellular proteins were precipitated with trichloroacetic acid and lysate samples were prepared in accordance with the manufacturer’s instructions for SulfoBiotics Protein Redox State Monitoring Kit Plus (Dojindo). The Protein-SHifter Plus that covalently binds to reduced protein thiols was added and the samples were subjected to electrophoresis through a 12% polyacrylamide gel. Each Protein SHifter Plus causes ~15 kDa shift of the protein bands. After electrophoresis, the gel was exposed to UV irradiation to excise the Protein-SHifter Plus moiety, and then subjected to electrotransfer to a nitrocellulose membrane and Western blotting with the Prx6 antibody. ( **A**) Representative Western blotting image of six experiments. ( **B**) Diagram of the native 25 kDa Prx6 molecule, the 40 kDa Prx6 molecule with one Protein-SHifter attached, and the 55 kDa Prx6 molecules with two Protein-SHifters attached. ( **C**) The bar graph represents means (± SEM) of the intensity of the 55 kDa band (N = 5). The symbol (*) denotes that the value is significantly different from all other values.

The technology developed for SulfoBiotics Protein Redox State Monitoring Kit Plus, by Dojindo Molecular Technologies adds a 15 kDa Protein-SHifter on free sulfhydryl groups, allowing the visualization of the thiol status of a given protein by coupling with immunoblotting. The human Prx6 molecule with a molecular weight of 25 kDa has two cysteine residues. Our results indicated that untreated human pulmonary artery smooth muscle cells predominantly contain the 55 kDa species, consistent with the Prx6 molecule, which has two Protein-SHifters incorporated, indicating that both cysteine residues occur in the reduced form in the cells ( Figure 1A, lane 1). Treatment of cells with PDGF (10 ng/ml) for 10 min, which promoted protein carbonylation of Prx6 ( Wong *et al.*, 2013), did not alter the thiol state of Prx6 ( Figure 1A, lane 1 and lane 2). The PDGF treatment for 30 min did not alter the thiol state of Prx6 either ( Figure 1A, lane 1 and lane 3). By contrast, treatment of H _2_O _2_ at a high concentration (1 mM) eliminated the 55 kDa band and generated a 40 kDa band that is consistent with one sulfhydryl group being oxidized ( Figure 1A, lane 4). These results were reproduced at least five times. Dataset 1 ( Suzuki *et al.*, 2017) contains the uncropped version of Figure 1A and the uncropped repeats. The bar graph shows the data from five separate experiments with five separate cell treatments. Control experiments were performed to ensure that PDGF stimulated protein phosphorylation as well as carbonylation.

The uncropped version of Figure 1A and the uncropped repeats Click here for additional data file. Copyright: © 2017 Suzuki YJ et al. 2017 Data associated with the article are available under the terms of the Creative Commons Zero "No rights reserved" data waiver (CC0 1.0 Public domain dedication).

## Discussion

Unlike protein carbonylation of Prx6, which is promoted in response to PDGF-treatment of human pulmonary artery smooth muscle cells ( Wong *et al.*, 2013), PDGF stimulation of cells does not cause the oxidation of two cysteine residues within the human Prx6 molecule. By contrast, cysteine oxidation within the Prx6 molecule can be promoted by treating cells with mM concentrations of H _2_O _2_ that are not likely to be generated in ligand/receptor-mediated cell signaling. We conclude that protein carbonylation, but not sulfhydryl oxidation, is a likely ROS-targeting mechanism for growth factor stimulation and cell signaling.

Protein carbonylation is promoted by metal-catalyzed generation of hydroxyl radicals, which are known to promote oxidation indiscriminately. However, the caged and site-directed production of hydroxyl radicals via metals could confer specificity ( Stadtman & Berlett, 1991; Wong *et al.*, 2010).

## Data availability

The data referenced by this article are under copyright with the following copyright statement: Copyright: © 2017 Suzuki YJ et al.

Data associated with the article are available under the terms of the Creative Commons Zero "No rights reserved" data waiver (CC0 1.0 Public domain dedication). 

**Dataset 1. The uncropped version of Figure 1A and the uncropped repeats.**

**DOI,** 10.5256/f1000research.11296.d157362 ( Suzuki *et al.*, 2017)
