# Supplementary figures and images for: Cell signaling promoting protein carbonylation does not cause sulfhydryl oxidation: Implications to the mechanism of redox signaling
Source: F1000Res. 2017 Apr 10;6:455. [Version 1] doi: 10.12688/f1000research.11296.1 (PMC5437949; doi:10.12688/f1000research.11296.1)

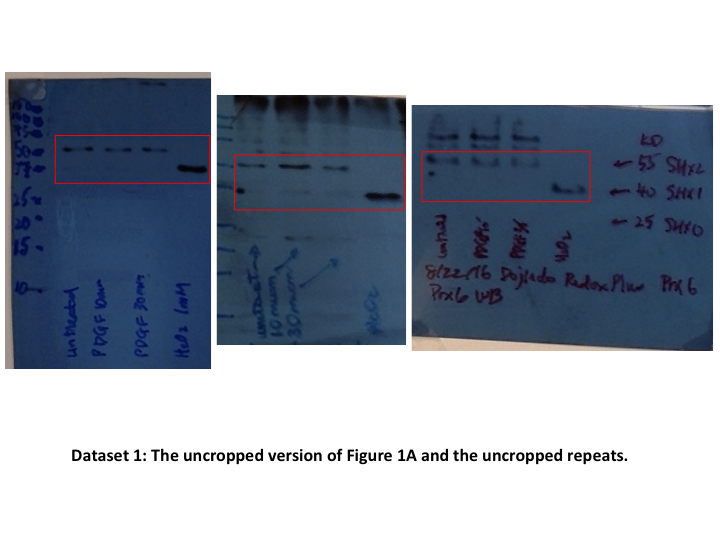

Supplement: The uncropped version of Figure 1A and the uncropped repeats [file f1000research-6-12189-s0000.tgz › 2417b169-c52b-451a-8e5f-3dbe9ff0f987_Suzuki_Dataset1_F1000Res_0317.tiff]
