# Supplementary material for: Cell signaling promoting protein carbonylation does not cause sulfhydryl oxidation: Implications to the mechanism of redox signaling
Source: F1000Res. 2017 Apr 10;6:455. [Version 1] doi: 10.12688/f1000research.11296.1 (PMC5437949; doi:10.12688/f1000research.11296.1)
Supplement: Peer review history

# Peer review: Referee response for version 1

**DOI:** 10.5256/f1000research.12189.r22633

**Published:** 2017-05-15

**Authors:** Brian McDonagh

**Affiliations:** 1 Department of Physiology, NUI Galway, Galway, Ireland

The authors describe the effects of PDGF and H2O2 treatment on the oxidation state of Prdx6 using a thiol probe, that when attached to free thiols increases the molecular weight of the protein by 15 kDa for each probe attached to the protein. The authors demonstrate that H2O2 treatment causes a change in the redox status of Prdx6 as compared to PDGF treatment. There are a number of issues that need to be resolved and validated by the authors before they can make some of the statements made within the manuscript.

 It is essential that the authors fully describe the sample preparation before analysis as this could greatly affect the results and interpretations made. In Figure 1 the authors describe that Prdx6 when the “Protein-SHifter” is added the protein has a mol weight of 55 kDa in controls and the PDGF treatments and one free thiol with the H2O2 treatment, but in Fig1B they show the native state of Prdx6 forming an intra- disulphide, was a reducing agent used in the sample preparation to reduce this disulphide? Does the catalytic Cys47 of this 1-Cys peroxiredoxin form an intra-disulphide with Cys91? It would also be helpful if a non “Protein-SHifter” treated sample was included in the blot to demonstrate the native band at 25 kDa. From Fig1A it would appear that there is a much more intense band for Prdx6 in the H2O2 treated samples, is there a loading control that can be included for this blot?

 Carbonylation usually refers to the introduction of an aldehyde or ketone group on an amino acid, I am not sure if this is what the authors are referring to in the title and throughout the manuscript. It is well known that Cys47 of Prdx6 forms a sulphinic (-SO2H) and/or sulphonic (-SO3H) acid. Indeed Prdx6 has been described as having quite a number of various modifications (Jeong, J et al, Proteomics, 2012) so the authors need to confirm the carbonylation or other modifications by mass spectrometry. It is clear that one of the Cys residues is not amenable to “Protein-SHifter” after H2O2 treatment, it would be helpful if they could identify which cysteine residue is susceptible to oxidation.

I have read this submission. I believe that I have an appropriate level of expertise to confirm that it is of an acceptable scientific standard, however I have significant reservations, as outlined above.

# Peer review: Referee response for version 1

**DOI:** 10.5256/f1000research.12189.r22403

**Published:** 2017-05-02

**Authors:** Tanea T. Reed

**Affiliations:** 1 Department of Chemistry, Eastern Kentucky University, Richmond, KY, USA

The authors studied the response of the antioxidant protein, peroxiredoxin-6 to treatment with PDGF and hydrogen peroxide. By using a commercially available kit, the authors discovered oxidation in one of the cysteine residues at high concentrations of H2O2. My only issue with this work is for Figure 1A. The authors state that they tested three time points of hydrogen peroxide, but only one is shown in the figure. By showing all three time points could further verify the finding of this report as the 40kD would be most potentially pronounced at 30  min.

I have read this submission. I believe that I have an appropriate level of expertise to confirm that it is of an acceptable scientific standard.

# Peer review: Referee response for version 1

**DOI:** 10.5256/f1000research.12189.r22184

**Published:** 2017-04-25

**Authors:** Sabah N.A. Hussain

**Affiliations:** 1 Meakins-Christie Laboratories, Translational Research in Respiratory Diseases Program, Department of Critical Care Medicine, Research Institute of the MUHC, McGill University Health Centre (MUHC), Montreal, QC, Canada

The authors provided indirect evidence that peroxiredoxin-6 does not undergo sulfhydryl oxidation when human pulmonary artery smooth muscle cells are exposed to PDGF but this protein undergo sulfhydryl oxidation when these cells were exposed to H _2_O _2_. It was concluded that protein carbonylation is more sensitive target of ROS during ligand/receptor-mediated cell signaling than sulfhydrul oxidation.

 Major comments: I believe that the conclusion of this study is too general and the authors should restrict themselves to the main findings of this study and do not extend their observation beyond one type of cells exposed to one growth factor (PDGF). 

 In addition, the authors used an indirect method to assess sulfydryl oxidation rather than a direct measurement. Moreover, the authors did not provide evidence in the current study that PDGF actually produced carbonylation of Prx6. This data is required to document the differential oxidation response of this protein to these two interventions (H2O2 vs. PDGF exposure).

 Finally, the authors need to provide data as to the time course of Prx6 oxidation in response to H2O2 exposure. They have only shown one time point.

I have read this submission. I believe that I have an appropriate level of expertise to confirm that it is of an acceptable scientific standard, however I have significant reservations, as outlined above.
